# Factors Related to the Performance of Elite Young Sailors in a Regatta: Spatial Orientation, Age and Experience

**DOI:** 10.3390/ijerph18062913

**Published:** 2021-03-12

**Authors:** Israel Caraballo, Alejandro Lara-Bocanegra, M. Rocío Bohórquez

**Affiliations:** 1 GALENO Research Group, Department of Physical Education, Faculty of Education Sciences, University of Cádiz, Cádiz, 11519 Puerto Real, Spain; israel.caraballo@uca.es; 2 Research Unit, Biomedical Research and Innovation Institute of Cádiz (INiBICA), 11519 Puerto Real, Spain; 3 Department of Physical Activity and Sport, Faculty of Education, University of Seville, 41013 Seville, Spain; 4 Department of Social Psychology, Faculty of Psychology, University of Seville, 41013 Seville, Spain; rociobohorquez@us.es

**Keywords:** spatial orientation, performance, sport sailing, special ability

## Abstract

The objective of this study was to examine the role of spatial orientation in the performance of sport sailors. Participants were 30 elite male sailors from classes 420, Laser, Windsurfing RS:X and Windsurfing Techno, grouped into two categories: Monohull (18 sailors) and Windsurfing (12 sailors). Ages ranged between 13 and 18 years old (M = 15.7, SD = 1.05). To assess spatial orientation, the Perspective Taking/Spatial Orientation Test was used, and performance was inferred from the final classification at the regatta. In addition, the influence of experience and age on the performance was analyzed. The results show that in the Monohull group, the performance is determined by the spatial orientation (18% of the explained variance), while in the Windsurfing group, the variables that are related to performance are sailing experience and age (60% of the explained variance). Spatial orientation seems to be the more important variable for performance in the Monohull group, while in classes belonging to the Windsurfing group, this variable does not seem to be decisive for obtaining good results in the regatta.

## 1. Introduction

Research in sports science has been extensively concerned with the study of the factors that influence performance in the different sports modalities. At present, the most developed lines of research in sailing are the influence of physiological, anthropometric, biomechanical, and training factors [1].

Sports performance is also determined by psychological training, so that, through the evaluation of psychological skills in the athlete, it is possible to predict their potential for success [2,3]. According to Olmedilla, Ortega, González and Serpa [4], sailing is a complex sport and psychological skills are key in learning this sport discipline. Authors such as Araújo, Davids and Serpa [5] or Brandt, Da Silva, Segato and Andrade [6] state that the most important psychological skills in this sport are attention span and decision making, and both skills are directly related to navigation tactics. Studies such as those carried out by Araújo and Serpa [7], Manzanares [8], and Manzanares, Menayo and Segado [9] show that elite sailors have a better visualization capacity and a greater number of fixations in relevant stimuli compared to amateur sailors. These two characteristics allow them to reduce the time taken to obtain relevant information, so they increase their chances to place themselves in optimal places, both at the exits and during the journey made [7,8,9]. In sport sailing, including all types of boats, perceptual skills are highly required, and this is mainly due to its high complexity. The sailor must anticipate his rivals and capture as much information as possible from them to carry out the most appropriate actions according to each circumstance [10]. The ability of anticipation seems to be a fundamental element within sports sailing because the environment in which it develops is characterized by being highly unstable. This instability is produced both by the weather conditions and by the different actions carried out by opponents. It must be taken into account that the sailor obtains the information to orient himself in a dynamic way; in addition, competitors become a dynamic reference because they are also at continuous movement, and buoys are the only static referents, making the action of orientation even more complex [11]. The sources of information that will determine the sailor’s orientation are the virtual representation of their position in space (rival sailors and buoys) and the cognitive map [12]. Through the cognitive map, the sailor is able to internally construct the context that surrounds him, thus being able to anticipate everything that is outside his visual field; this allows a more correct programming of the route to be followed [13]. Authors like Gómez, Rousset and Baciu [14] and Montello [15] affirm that a continuous updating of the spatial information received from the environment is necessary, because it allows the subject to be aware of their current position at all times. While the subject performs the task, the orientation process is automatic, but when the information is updated, the effort made by the athlete increases [16].

For Maier [17] the main components of spatial ability are spatial rotations, spatial perception, spatial visualization, mental rotation and spatial orientation. In spatial rotation, the reference frame used can be intrinsic or relative to an object; this frame of reference is defined by the upper/lower, anterior/posterior and right/left intrinsic axes [18].

Spatial perception, the information processing approach, has mainly been used to analyze spatial skills on a small scale; these skills use the speed of encoding and transformation of spatial information, memory capacity and spatial work strategies as differentiation elements [19,20]. Spatial visualization is more complex than relationship or orientation tasks; this circumstance occurs because the tasks that comprise it have a spatial figurative component in which movement or displacement of the figure’s elements occurs [21]. Mental rotation is defined as the ability to rotate figures quickly and accurately in the mind [22]. Spatial orientation refers to the subject’s ability to have a different perspective on an object when the observer is redirected [23]. Different systems for evaluating spatial ability have been used, although the most widespread are those that differentiate between large-scale and small-scale spatial abilities proposed by Ittelson [24], and endorsed and specified by Voyer, Voyer and Bryden [25]. Despite this differentiated classification, research demonstrates that small-scale spatial abilities predict performance on large-scale tasks, because spatial abilities at different scales are partially but not totally dissociated [26,27,28].

Spatial orientation—a large-scale ability—is the most commonly used component for the evaluation of spatial ability and is considered the most important component [29,30]. The literature on spatial orientation often differentiates between egocentric and allocentric representations, also called exocentric or geocentric [31]: egocentric representations are self-to-object-centered, while the allocentric anchor on environmental information uses object-to-object references. Recent studies have shown that both are related, since egocentric sensory information can be transformed into allocentric representations [32]; specifically, some studies affirm that the performance of the subjects in orientation capacity, in relation to the different elements that surround them, are related to the score they obtain in perspective-taking tasks [33,34]. For various tasks, navigators profit from both kinds of representation [35].

Regarding to experience, small differences in time of practice can make a huge difference, as is well known in the expertise literature [36,37]. The influence on performance of a multitude of personal (time of practice, physiognomy), social (influence of third parties) and contextual (culture, access to practice) factors has been postulated. Specifically, athletic preparation is capable of developing spatial ability [38], so athletes with more experience are able to extract critical information from a greater number of spatial references than novices [39]. Specifically, sailing experience has proven to be decisive for the development of strength, resistance and speed-oriented motor coordination and, therefore, for better sports performance [1,40,41]. In this sense, but in relation to psychological variables, experienced sailor decision-making is characterized by the cumulative non-linear effects of exploring and using information constraints in a regatta, making their performance better than the less experienced sailors [5]. Regarding the indicators of attention management, the time of recurrence of the fixation of the gaze is less in the less experienced sailors, whether they are relevant or irrelevant stimuli for the regatta [42]. Related to spatial ability, experience was shown to significantly influence performance in specific tests [43].

Age has not always been shown to generate differences in the spatial skills of the general population: sometimes, it has been postulated as a consequence of experience in different tasks or contexts [44], while, other times, it has been pointed out that the maturation of evolutionary development generates differences in spatial ability [45]. Those authors who propose that age influences the development of spatial orientation skills only because of its relationship with experience demonstrated that experts, unlike novices, showed a progression towards the extraction of more information early as a function of age [38,39], although it was only at the adult level that the anticipatory performance of the expert players significantly outperformed that of their novice counterparts (Abernethy). On the other hand, other authors have postulated a direct effect of age on the development of spatial orientation: the literature suggests that 3- to 10-year-old children develop their ability to combine egocentric and allocentric forms of spatial coding, and show adult-level performance on cognitive mapping tasks at around 10 or 12 years of age [45,46]. Regarding the decline in spatial orientation skills, some studies found that adults between 46 and 67 years of age performed worse than younger participants (18–30 or 31–45 years) in all the orientation skills evaluated [47], while other investigations pointed out that the egocentric spatial orientation progressively improves from 8 to 60 years old [48]. To our knowledge, the relationship between age and performance in spatial orientation tasks hasn’t been studied with sport sailors.

Following what has been developed in the literature, the main objective of this study is to verify whether spatial orientation, experience and age are related to the performance of the sailors in a regatta. The following hypotheses emerge from this objective:

**Hypothesis** **1.** **(H1).** *The better the spatial orientation of the sailors, the better performance they will obtain in competition.*

**Hypothesis** **2.** **(H2).** *The more years of sailing experience, the better performance sailors will obtain in competition.*

**Hypothesis** **3.** **(H3).** *The age of the sailors will not allow predicting their performance in competition.*

## 2. Materials and Methods

### 2.1. Participants

The investigation involved 30 elite sailors belonging to classes 420, Laser, Windsurfing RS:X and Windsurfing Techno which took part of the competition circuit of the Andalusian Sailing Federation (Spain) and participated regularly in national and international competitions. All sailors were male and their ages ranged from 13 to 18 years (M = 15.70, SD = 1.06). Regarding their sportive experience, they have been sailing between 2 and 11 years (M = 6.50, SD = 2.78).

### 2.2. Instruments

A sociodemographic questionnaire was designed including questions related to age, sex, class to which they belonged and years of experience in sailing.

**Figure 1:**
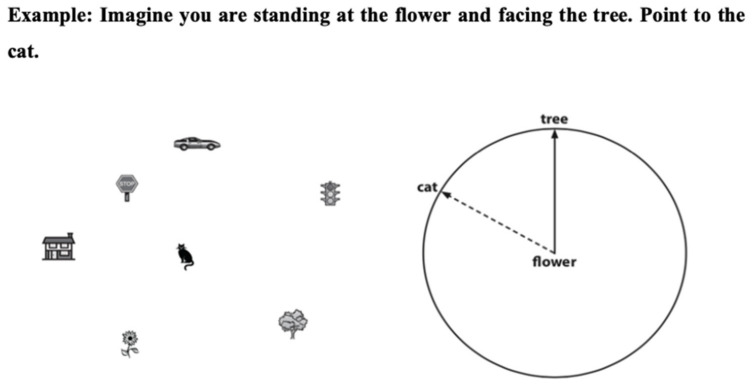
Spatial orientation test.

The test designed by Kozhevinikov and Hegarty [21] was used to evaluate spatial orientation, as a modified version of Hegarty and Waller [23]. It includes 12 items in paper and pencil format (Figure 1). Each reactive is composed of a matrix with seven objects and a circle in which one direction is marked; participants should imagine their position in the circle and mark the direction towards one of the seven objects determined in the question. The result of each question is calculated by the absolute deviation in degrees between the response of the participant and the correct address of the assigned object. Each reactive may be scored between 0 and 180°. To obtain the final score, the average deviation of the 12 items must be calculated. The items that were not answered were assigned a value of 90°. To finish the test, there is a maximum time of five minutes. The established reliability for this test was 0.83 in its original formulation [49].

### 2.3. Procedure

Two weeks before the competition, the Andalusian Sailing Federation and the coaches of the different sailing clubs that participated in the regatta were contacted and informed about the objectives of the study, and invited to participate voluntarily. The day before the competition, a room was set up at the headquarters of the Andalusian Sailing Federation in which the tests were realized.

All sailors who participated voluntarily in this study had to sign an authorization form. Participants under 18 also required authorization from their parents and/or guardians by signing informed consent.

### 2.4. Regatta

To assess the performance, results from the final ranking at the XV New Year´s Race “X Memorial Kim Lythgoe” were used [50]. The ranking was determined with the sum of the positions obtained in each of the tests, in which a better classification meant a lower result in the final ranking. The race was held at the headquarters of the Specialized Center for Sailing Sports Technification “Bahía de Cádiz” from 27–30 December 2018. This regatta is of an international category. In the evaluated classes, a total of 83 sailors competed, so the study represents 31% of the total participants in the competition in classes 420 (14 sailors), Laser (38 sailors) and windsurfing (38 sailors). During the competition days, the 420 class made a total of four races and the Laser and Windsurfing classes performed six. In each length of the race, sailors complete a tour that was previously established by the organizing committee.

### 2.5. Data Analysis

The data obtained were reviewed for outliers and other anomalies; no adjustment or modification of the database was necessary. After that, two groups of sailors were created: classes 420 and Laser were united in a group called Monohull, and classes RS:X and Techno joined the group called Windsurfing. The justification for dividing these boats into two groups is because the boats that belong to each group share a series of similar characteristics in their structure as in their handling; however, these characteristics are totally different from the other group. In addition, the Windsurfing class, due to their characteristics, can develop faster than the monohull group boats; therefore, the way the racecourse is organized is very different between the classes that belong to each group.

Linear regression analyses were performed for Monohull and Windsurfing classes to examine the association between spatial orientation, experience, age and performance. The method used to perform the linear regression analysis was Stepwise in order to find the strongest predictor for ranking. To check the prediction value of the performance of the equations obtained, the Student’s *t*-test was performed between the variables’ real ranking and predicted ranking with this equation.

## 3. Results

Descriptive analysis and the results obtained in the spatial orientation test by the sailors of the Monohull and Windsurfing classes are shown in Table 1.

Table 2 shows the linear regression analysis. We obtain a model in one step to find the optimal model, which has a linear relationship of 47% and a goodness of the fit = 0.18. This model includes the constant and the score variable in the spatial orientation test, excluding the navigation experience and age variables. The value of Durbin–Watson (1.51) indicates that our error variance is independent; therefore, it would be appropriate to use a linear model. The multicollinearity analysis shows high tolerance values in the variable spatial orientation test (0.88), the years of navigation experience (0.90) and the age (0.83). These results indicate that these variables do not show collinearity. The equation obtained with this model for the prediction of performance is the following: performance = 20.48 + (−0.20 * orientation test). When checking the value of the prediction of the performance obtained with this equation, using the Student’s *t*-test to compare the relationship between the ranking variable and the ranking variable estimated, we observe that the error was −0.01.

**Figure 2:**
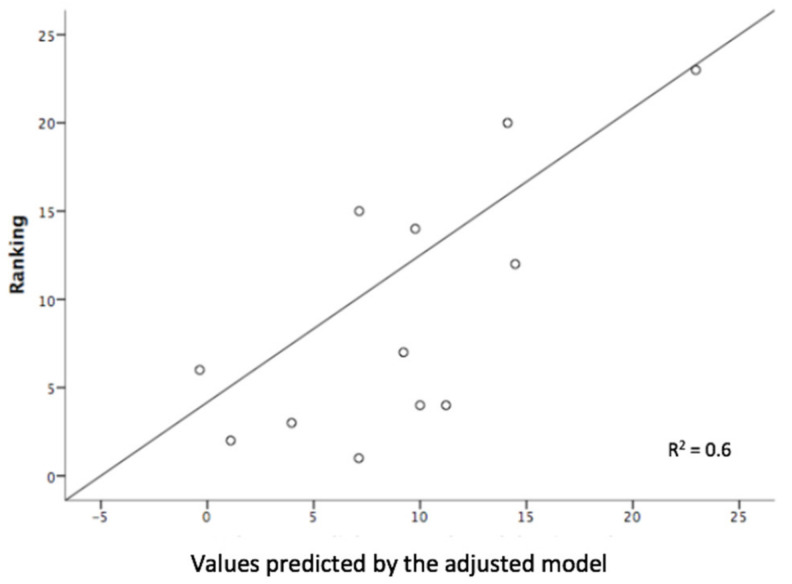
Fitted linear regression graph of model 2 for the Windsurfing group. Method of successive steps. Variables: age and sailing experience.

In the linear regression analysis for the Windsurfing group, we obtain two models in two steps and choose Model 2, which has a linear relationship of 82% and a goodness of 0.6 fit (Figure 2). This model excludes the spatial orientation test variable but includes the constant and the navigation experience and age variables of the sailor (Table 2). It would be appropriate to use this model since the value of Durbin–Watson (2.29) indicates that the residuals are independent. The most important variable in this model would be the age, since it contributes 83%, while the navigation experience would contribute 50%. The high tolerance values in the multicollinearity analysis of the variable spatial orientation test (0.95), the years of navigation experience (0.88) and the age (0.861) indicate that there is no collinearity for these variables.

The equation obtained to predict the performance in the Windsurfing group would be determined as follows: performance = −104.68 + (7.72 * Age) + (−1.23 * Sailing experience). Checking the prediction value of this equation indicates that the error made is very low, its value being 0.00.

## 4. Discussion

The main objective of this study was to verify whether the spatial orientation, experience and age are related to the performance of the sailor in a regatta. First, it was hypothesized that the better the spatial orientation of the sailors, the better their performance in competition. The results only allow us to partially accept this hypothesis, since the spatial orientation predicted the performance of the sailors of the Monohull group, but not of the Windsurfing group. The correlation between spatial orientation and performance found in Monohull was also described with sailors of the Optimist class by Manzanares [8]. The spatial ability–performance relationship is based on the need of sailors to anticipate the behavior of their rivals [10], as well as the influence of environmental factors at each moment of the regatta [11], aspects directly related to performance in sailing, allowing a competitive advantage to be exploited [7,8,9]. It is possible to infer that both large- and small-scale spatial skills are involved in the sports behavior of sailors, and that they are closely related to each other [26,27,28] and to performance in real outdoor tasks [35]. We must also consider that, during the competition, sailors must pay attention to a visual field in motion and this may interfere with the cognitive tasks he is performing. Therefore, if the subject has a good spatial orientation, their attention resources will not be diverted by the movement that occurs in the objects of their visual field, thus they will not lose cognitive resources that attend to postural control and could affect the execution of sports technical gestures [51].

A priori, spatial orientation should be important for both Monohull and Windsurf groups, but our results only confirm its influence on the Monohull group. It is possible that the characteristics of each of these groups, and the navigation peculiarities that characterize them, may explain the differences found between both groups [52]. The sailors of the Windsurfing class, due to the difference between the large size of the sail surface and the reduced length/beam of the boat, perform a specific action after each turn to recover the speed, which is very efficient when the wind speed is less than fifteen knots, and is called rowing with the sail (sail-pumping) [53]. Monohull-type vessels, such as 420 and Laser, do this at very specific times, but, in the windsurfing class, it is done both when it goes against the wind and when it goes upwind, although when it is against the wind the physical demands on the Sailor are higher [54]. Therefore, it is logical to think that the lower the number of maneuvers performed during the tour, the lower the number cognitive demands required. However, we do not have information to ensure those aspects, so future research should address them and other possible hypotheses.

The second hypothesis predicted that the more years of sailing experience, the better sailors’ performance in competition. The results prevent us from fully accepting this hypothesis, since experience allowed us to predict the performance of athletes in the windsurfing group, but not in the monohull group. The influence of experience on performance has been demonstrated both in the general literature [36,37] and the literature specific to navigation [5,42,43]. It is likely that the most expert sailors have a greater orientation, which is demonstrated in a better analysis of the positions of the buoys in the racecourse and the route chosen to approach them [55], as well as their ability to obtain the most relevant information on the racecourse and thus be able to execute efficient motor actions according to the characteristics of the situation [56,57,58]. The most experienced sailors were placed in a better position with respect to their rivals to obtain a more favorable wind and reduce the number of maneuvers they had to perform when exceeding the buoy. This tangent point seems to be critical when it comes to rounding on the windward buoy, and a bad choice would make the maneuvers come forward or backward with respect to the maneuvers performed by their opponents, with expert sailors reacting differently compared to inexperienced sailors [55]. Another aspect related to the spatial orientation is the perception of the wind direction and the orientation that the boat has with respect to this, which is key when differentiating between expert sailors and beginners [59]. That differentiation in the orientation with respect to the wind is more prominent in situations in which the wind speed is weak, highlighting even more the sailors with great experience [60]. Moreover, indirect effects of experience on performance are possible: having more experience could benefit the orientation skills, making the sailors more comfortable in general in those situations in which this skill is required, and therefore obtaining better sport performance [43].

Despite that general agreement, our results only confirm the hypothesis for the Winsurf group. We do not have information that allows us to create hypotheses about the origin of this contradictory result, so future studies should address this problem by accessing both personal, social and contextual variables.

Finally, it was hypothesized that the age of the sailors would not allow for prediction of their performance in competition. This hypothesis is accepted only for the Monohull group; for the Windsurf group, age—alone or in combination with experience—makes it possible to predict performance in the regatta. From the theoretical perspective, which defends that age influences spatial ability, this result is consistent with that provided by Ruggiero et al. [48], who found that the egocentric spatial orientation progressively improves from 8 to 60 years old as a consequence of maturation [35]. For our Windsurf sailors, older age probably implies better ability to visualize, allowing for the most relevant information of the competition environment and, therefore, better performance [61].

We do not have a hypothesis that allows us to explain or suppose why age influences the performance of Windsurfers, but not that of Monohulls. The sailors in both groups are homogeneous in terms of age and experience, so these variables could act in the same way in both groups. Some personal, social or contextual variables of these groups must mediate the relationship between age and performance. Future research should delve into these possible modulating variables.

In our study, we can consider some limitations. Only one test was used in this study to assess spatial orientation; it would have been very interesting to obtain data from a Mental Rotation test or Spatial Anxiety Scale to compare and corroborate our results. In the same way, performance was measured by a single event, so it is necessary to assume that it may not reflect the average performance of the sailors, but only that of this test. Moreover, due to the small number of the sample, it is not possible to extrapolate these results to the sailor population. However, to our knowledge, this is the first study analyzing the influence of spatial orientation ability, age and experience on sailors´ performance when belonging to different classes.

## 5. Conclusions

The results of this study demonstrate that, for sailors in the Monohull group (classes 420 and Laser), spatial orientation predicts competitive performance in a given regatta. In addition, for the athletes of the Windsurf group (classes RS:X and Techno), both the sailing experience and the age, and the combination of both, allow for prediction of the performance in a certain competition. Thus, it seems that, between both groups of navigators, there are personal, social or, more likely, contextual differences that explain dissimilar results.

## Figures and Tables

**Table 1:** Participants’ age, experience and score obtained in the Spatial Orientation Test by groups and classes.

	Monohull			Windsurfing		
	All (*n* = 18)	420 (*n* = 6)	Laser (*n* = 12)	All (*n* = 12)	RS:X (*n* = 5)	Techno (*n* = 7)
Sailing experience (years)	7.33 ± 2.35	6.83 ± 1.94	7.58 ± 2.57	5.25 ± 2.99	6.20 ± 3.35	4.57 ± 2.76
Age (years)	15.78 ± 1.22	15.17 ± 0.75	16.08 ± 1.31	15.58 ± 0.79	16.40 ± 0.55	15.00 ± 0.00
Spatial Orientation Test	35.12 ± 22.98	50.03 ± 31.75	27.66 ± 13.25	60.04 ± 50.08	62.86 ± 60.89	58.03 ± 45.98

Note: Data presented in Mean ± SD.

**Table 2:** Coefficients of the linear regression models in Monohull and Windsurfing groups.

Model		Non-Standardized Coefficients	Typified Coefficients	t		Sig.	Confidence Interval 95% for B	
		B	Std. Error	Beta			Lower Bound	Upper Bound
Monohull	Constant	20.48	3.94		5.19	0.00 **	12.12	28.84
	S. O. T.	−0.20	0.09	−0.47	−2.16	0.04 *	−0.40	−0.00
Windsurfing	Constant	−88.19	33.96		−2.59	0.02 *	−163.87	−12.51
	Age	6.25	2.17	0.67	2.87	0.01 *	1.40	11.10
	Constant	−104.68	28.21		−3.71	0.00 **	−168.51	−40.85
	S. E.	−1.23	0.49	−0.50	−2.51	0.03 *	−2.35	−0.12
	Age	7.72	1.85	0.83	4.16	0.00 **	3.53	11.92

Note: S. O. T. = Spatial orientation Test; S. E. = sailing experience. Method used: “Stepwise”. ** *p* < 0.01; * *p* < 0.5.

## Data Availability

No new data were created or analyzed in this study. Data sharing is not applicable to this article.

## References

[1] Callewaert M., Boone J., Celie B., De Clerq D., Bourgois J.G. (2015). Indicators of sailing performance in youth dinghy sailing. Eur. J. Sport Sci..

[2] Gould D., Maynard I. (2009). Psychological preparation for the Olympic games. J. Sports Sci..

[3] Serpa S., Castro T. (2006). Psychology of the Olympic Games: The perception of coaches. J. Sport Psychol..

[4] Olmedilla A., Ortega E., González J., Serpa S. (2015). Psychological training in sailing: Performance improvement for the Olympic classification phase. Univ. J. Psychol..

[5] Araújo D., Davids K., Serpa S. (2005). An ecological approach to expertise effects in decision-making in a simulated sailing regatta. Psychol. Sport Exerc..

[6] Brandt R., Da Silva M., Segato L., Andrade A. (2012). Atençâo em velejadores conceitos e aplicaçâo. Rev. Bras. Ciências Esporte.

[7] Araújo D., Serpa S. (1999). Dynamic decision making at different levels of experience in sailing sport. J. Sport Psychol..

[8] Manzanares A. (2013). Analysis of Visual Behavior and Performance Achieved at the Star of Simulated Races: Differences Base on Experience. Ph.D. Thesis.

[9] Manzanares A., Menayo R., Segado F. (2017). Visual search strategy during regatta starts in a sailing simulation. Motor Control.

[10] Ward P., Williams A.M., Bennett S. (2002). Visual search and biological perception in tennis. Res. Quart. Sport Exerc..

[11] Anne L. (1990). Spatial orientation skills and mathematical problem solving. J. Res. Math. Educ..

[12] Wolbers T., Wiener J.M. (2014). Challenges for identifying the neural mechanisms that support spatial navigation: The impact of spatial scale. Front. Hum. Neurosci..

[13] Boccia M., Piccardi L., D´Alessandro A., Nori R., Guariglia C. (2017). Restructuring the navigational field: Individual predisposition towards field independence predicts preferred navigational strategy. Exp. Brain Res..

[14] Gómez A., Rousset S., Baciu M. (2009). Egocentric-updating during navigation facilities episodic memory retrieval. Acta Psychol..

[15] Montello D.R., Shan P., Miyake A. (2005). Navigation. The Cambridge Handbook of Visuospatial Thinking.

[16] Wang R., Brockmole J. (2003). Simultaneous spatial updating in nested environments. Psychon. Bull. Rev..

[17] Maier P., Osnabrück E., Cohors-Fresenborg K., Reiss G., Toener G., Weigand H., Verlag-Franzbecker K.G. (1998). Spatial Geometry and Spatial Ability: How to Make Solid Geometry Solid. Selected Papers from the Annual Conference of Didactics of Mathematics.

[18] Wraga M., Creem S., Proffitt D. (1999). The influence of spatial reference frames on imagined object and viewer rotations. Acta Psychol..

[19] Hegarty M., Waller D., Shah P., Miyake A. (2005). Individual differences in spatial abilities. The Cambridge Handbook of Visuospatial Thinking.

[20] Just M.A., Carpenter P.A. (1985). Cognitive coordinate systems: Accounts of mental rotation and individual differences in spatial ability. Psychol. Rev..

[21] Kozhevnikov M., Hegarty M. (2001). A dissociation between object manipulation spatial ability and spatial orientation ability. Mem. Cogn..

[22] Hines M., Fane B.A., Pasterski V.L., Mathews G.A., Conway G.S., Brook C. (2003). Spatial abilities following prenatal androgen abnormality: Targeting and mental rotations performance in individuals with congenital adrenal hyperplasia. Psychoneuroendocrinology.

[23] Hegarty M., Waller D. (2004). A dissociation between mental rotation and perspective-taking spatial abilities. Intelligence.

[24] Ittelson W.H. (1973). Environment and Cognition.

[25] Voyer D., Voyer S., Bryden M.P. (1995). Magnitude of Sex Differences in Spatial Abilities: A Meta-Analysis and Consideration of Critical Variables. Psychol. Bull..

[26] Hegarty M., Burte H., Boone A.P., Montello D.R. (2008). Individual differences in large-scale spatial abilities and strategies. Handbook of Behavioral and Cognitive Geography.

[27] Hegarty M., Montello D.R., Richardson A.E., Ishikawa T., Lovelace K. (2006). Spatial abilities at different scales: Individual differences in aptitude-test performance and spatial-layout learning. Intelligence.

[28] Wang L., Carr M. (2004). Working Memory and Strategy Use Contribute to Gender Differences in Spatial Ability. Educ. Psychol..

[29] Linn M., Petersen A. (1985). Emergence and Characterization of Sex differences in Spatial Ability: A Meta-Analysis. Child Dev..

[30] Mafalda R. (2000). Efeitos do uso de Diferentes Métodos de Representacao Gráfica no Desenvolmimento da Habilidade de Visualizacao Espacial. Ph.D. Thesis.

[31] Klatzky R.L., Freksa C., Habel C., Wender K.F. (1998). Allocentric and Egocentric Spatial Representations: Definitions, Distinctions, and Interconnections. Spatial Cognition. Lecture Notes in Computer Science.

[32] Wang C., Chen X., Knierim J.J. (2020). Egocentric and allocentric representations of space in the rodent brain. Curr. Opin. Neurobiol..

[33] Fields A., Shelton A. (2006). Individual skill differences and large-scale environmental learning. J. Exp. Psychol..

[34] Kozhevnikov M., Motes M.A., Rasch B., Blajenkova O. (2006). Perspective-taking vs. mental rotation transformations and how the predict spatial navigation performance. Appl. Cogn. Psychol..

[35] Meilinger T., Vosgerau G., Hölscher C., Shipley T.F., Olivetti Belardinelli M., Bateman J.A., Newcombe N.S. (2010). Putting Egocentric and Allocentric into Perspective. Lecture Notes in Computer Science.

[36] Baker J., Farrow M. (2017). Routledge Handbook of Sport Expertise.

[37] Williams A.M., Ericsson K.A. (2005). Perceptual-cognitive expertise in sport: Some considerations when applying the expert performance approach. Hum. Mov. Sci..

[38] Abernethy B. (1988). The Effects of Age and Expertise upon Perceptual Skill Development in a Racquet Sport. Res. Q. Exerc. Sport.

[39] Notarnicola A., Maccagnano G., Pesce V., Tafuri S., Novielli G., Moretti B. (2014). Visual- spatial capacity: Gender and sport differences in young volleyball and tennis athletes and non-athletes. BMC Res. Notes.

[40] Castagna O., Brisswalter J. (2007). Assessment of energy demand in Laser sailing: Influences of exercise duration and performance level. Eur. J. Appl. Physiol..

[41] Vangelakoudi A., Vogiatzis I., Geladas N. (2007). Anaerobic capacity, isometric endurance, and Laser sailing performance. J. Sports Sci..

[42] Sjøgaard G., Inglés E., Narici M. (2015). Science in sailing: Interdisciplinary perspectives in optimizing sailing performance. Eur. J. Sport Sci..

[43] Devlin A.S. (2004). Sailing Experience and Sex as Correlates of Spatial Ability. Percept. Motor Ski..

[44] Schoenfeld R., Lehmann W., Leplow B. (2010). Effects of Age and Sex in Mental Rotation and Spatial Learning from Virtual Environments. J. Individ. Differ..

[45] Lehnung M., Leplow B., Friege L., Herzog A., Ferstl R., Mehdorn M. (1998). Development of spatial memory and spatial orientation in preschoolers and primary school children. Br. J. Psychol..

[46] Newcombe N.S. (2019). Navigation and the developing brain. J. Exp. Biol..

[47] Liu I., Levy R.M., Barton J.J.S., Iaria G. (2011). Age and gender differences in various topographical orientation strategies. Brain Res..

[48] Ruggiero G., D´Errico O., Iachini T. (2016). Development of egocentric and allocentric spatial representations from childhood to elderly age. Psychol. Res..

[49] Lin C.H., Chen C.M., Lou Y.C. (2014). Developing Spatial Orientation and Spatial Memory with a Treasure Hunting Game. Educ. Technol. Soc..

[50] XV New Year’s Race “X Memorial Kim Lythgoe”. https://andaluza.fav.es/default/events/event/text/xv-new-year’s-race-x-memorial-kim-lythgoe.

[51] Ehrenfierd T., Guerraz M., Thilo K.V., Yardley L., Gresty M.A. (2003). Posture and mental task performance when viewing a moving visual field. Cogn. Brain Res..

[52] Bojsen-Moller J., Larsson B., Magnusson P., Aagaard P. (2007). Yacht type and crew-specific differences in anthropometric, aerobic capacity, and muscle strength parameters among international Olympic class sailors. J. Sports Sci..

[53] Vogiatzis I., De Vito G. (2015). Physiological assessment of Olympic windsurfers. Eur. J. Sport Sci..

[54] Vogiatzis I., De Vito G., Radio A., Marchetti M. (2005). Comparison of the physiological responses to upwind and downwind sail-pumping in windsurfers. N. Zeal. J. Sport Med..

[55] Pluijms J., Cañal-Bruland R., Bergmann-Tiest W.M., Mulder F.A., Savelsbergh G.J.P. (2015). Expertise effects in cutaneous wind perception. Atten. Percept. Psychophys..

[56] Tenebaum G., Starkes J., Ericsson K. (2003). Expert athletes: An integrated approach to decision making. Expert Performance in Sport, Advances in Research on Sport Expertise.

[57] Vickers J. (2007). Perception, Cognition and Decision Training. The Quiet Eye in Action.

[58] Manzanares A., Menayo R., Segado F., Salmerón D., Cano J.A. (2015). A probabilistic model for analysing the effect of performance levels on visual behaviour patterns of young sailors in simulated navigation. Eur. J. Sports Sci..

[59] Prlenda N., Oreb I., Vujcic D. (2018). Buoys as a tool in teaching basic elements of sailing. Sportlogia.

[60] Pluijms J.P., Cañal-Bruland R., Hoozemans M.J., Savelsbergh G.J. (2015). Visual search, movement behavior and boat control during the windward mark rounding in sailing. J. Sports Sci..

[61] Newcombe N., Bandura M.M. (1983). Effect of age at puberty on spatial ability in girls: A question of mechanism. Dev. Psychol..

